# Supplementary material for: Real-time visualization of intratumoral necrosis using split-luciferase reconstitution by protein trans-splicing
Source: Mol Ther Oncolytics. 2020 Dec 10;20:48–58. doi: 10.1016/j.omto.2020.12.001 (PMC7851486; doi:10.1016/j.omto.2020.12.001)
Supplement: Document S1. Figures S1 and S2 and Supplemental Materials and Methods [file mmc1.pdf]

OMTO, Volume *20*

Supplemental Information

Real-time visualization of intratumoral necrosis using split-luciferase reconstitution by protein trans-splicing

Go Kagiya, Ayaka Sato, Ryohei Ogawa, Masanori Hatashita, Mana Kato, Makoto Kubo, Fumiaki Kojima, Fumitaka Kawakami, Yukari Nishimura, Naoya Abe, and Fuminori Hyodo

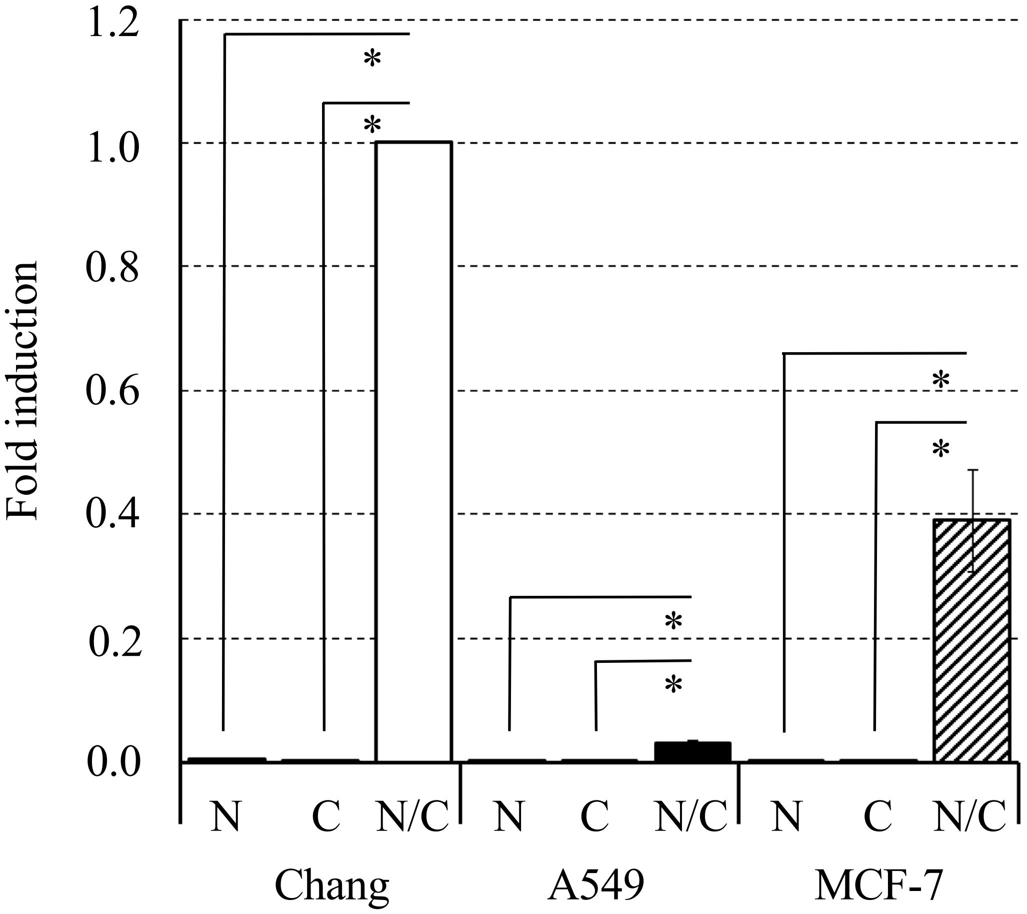

Figure S1. A comparison of the bioluminescence intensities obtained through the interaction of necrosis imaging reporters in three different cell lines. Chang liver cells, A549 cells and MCF-7 cells, transiently transfected with either pNLucN/v2 or pNLucC/v2-Fc were recovered at 12 h after transfection. The recovered cells were either lysed in a single transfectant or in a combination of the mutual transfectants of one cell line in PLB and then were incubated at an ambient temperature before undergoing the luc assay. Their bioluminescence values were normalized to those of the combined transfectant cell lysates with pNLucN/v2 and pNLucC/v2-Fc in Chang cells and then plotted as the mean fold induction. Error bars represent the standard deviation (n = 3). Asterisks indicate statistical significance ($p < 0.01$).

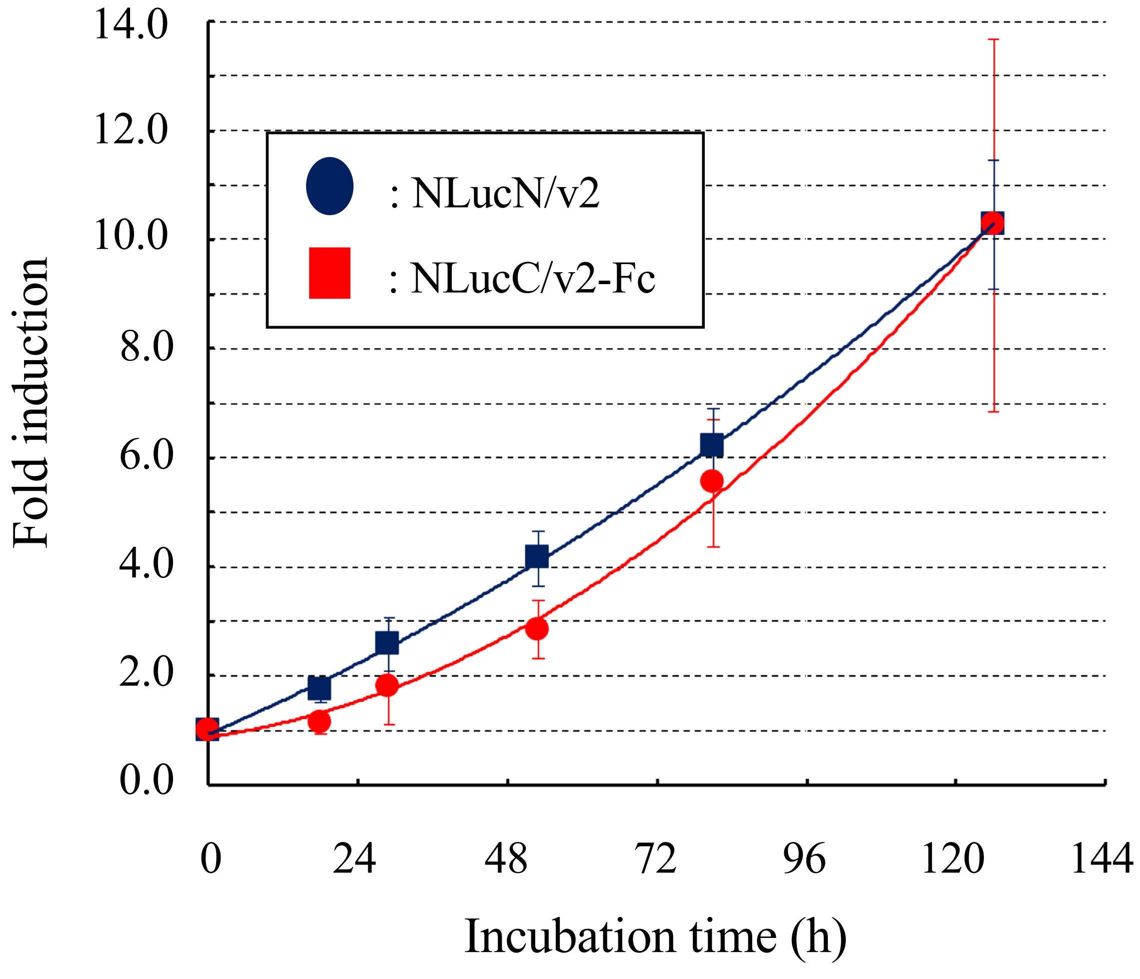

Figure S2. Cell proliferation curves of NLucN/v2 and NLucC/v2-Fc, which are stably transfected cell lines with pNLucN/v2 and pNLucC/v2-Fc, respectively. The cells inoculated in 96-well plate were pre-incubated at 37 ° C for 12 h, and then WST-8 was added to each well. The absorbance values obtained at 450 nm were normalized against those of the same cell line at the beginning point and then were plotted as the cell growth curve (n = 3).

Supplemental methods:

Cell proliferation measurement

The cell proliferation measurements were performed with the Cell Counting Kit-8 (Dojindo, Kumamoto, Japan) according to the manufacturer's instructions. Two necrosis imaging reporter cell lines, NLucN/v2 and NLucC/v2-Fc (2.0×10^3 cells/100 µl/well), were cultured in a 96-well plate for 12 h before 10 µl of WST-8, the colorimetric reagent of the kit, was added to each well and incubated at 37 °C for 2 h. The absorbance value of each well was determined at 450 nm using a Multiskan FC micro-plate reader (Thermo Fisher Scientific, Waltham, MA, USA). Since the relationship between the cell numbers and absorbance at 450 nm after the addition of WST-8 was confirmed to be linearly correlated, the absorbance ratios normalized against those of the same cell line at the beginning point were plotted as the cell growth curve.
