# Supplementary material for: Real-time visualization of intratumoral necrosis using split-luciferase reconstitution by protein trans-splicing
Source: Mol Ther Oncolytics. 2020 Dec 10;20:48–58. doi: 10.1016/j.omto.2020.12.001 (PMC7851486; doi:10.1016/j.omto.2020.12.001)
Supplement: Document S2. Article plus Supplemental information [file mmc2.pdf]

Molecular Therapy
Oncolytics
Original Article

Real-time visualization of intratumoral necrosis using split-luciferase reconstitution by protein trans-splicing

Go Kagiya,[1,2] Ayaka Sato,[3] Ryohei Ogawa,[4] Masanori Hatashita,[5] Mana Kato,[6] Makoto Kubo,[1,2] Fumiaki Kojima,[1,2] Fumitaka Kawakami,[1,2] Yukari Nishimura,[1] Naoya Abe,[1] and Fuminori Hyodo[7]

[1]School of Allied Health Sciences, Kitasato University, 1-15-1 Kitasato, Minami-ku, Sagamihara, Kanagawa 252-0373, Japan; [2]Regenerative Medicine and Cell Design Research Facility, Kitasato University, 1-15-1 Kitasato, Minami-ku, Sagamihara, Kanagawa 252-0373, Japan; [3]Department of Radiology, St. Marianna University School of Medicine, 2-16-1 Sugao, Miyamae, Kawasaki, Kanagawa 216-8511, Japan; [4]Department of Radiological Sciences, Graduate School of Medicine and Pharmaceutical Sciences, University of Toyama, 2630 Sugitani, Toyama 930-0194, Japan; [5]Biotechnology Division, The Wakasa Wan Energy Research Center, 64-52-1 Nagatani, Tsuruga, Fukui 914-0192, Japan; [6]Department of Radiological Services, Tokyo Women's Medical University Hospital, 8-1 Kawada-cho, Shinjuku-ku, Tokyo 162-8666, Japan; [7]Department of Radiology Frontier Science for Imaging, School of Medicine, Gifu University, 1-1 Yanagida, Gifu 501-1194, Japan

Necrosis, a form of cell death, occurs not only with the development of various diseases but also with a tumor tissue response to cancer treatment. Therefore, pursuing progress for cancer therapy through induction of necrosis may be one of the most effective approaches for cancer eradication. We herein describe the development of a real-time imaging system to visualize intratumoral necrosis. The system is composed of two types of cells expressing either one of two necrosis imaging reporters that consist of a DnaE intein sequence linking to one of two split-luciferase fragments. When necrosis occurs in a tumor composed of both of the cells, the two types of leaked reporters can reconstitute the enzymatic activity as a result of protein *trans*-splicing and thereby emit bioluminescence in the presence of the substrate. This system, which was constructed with shrimp-derived luciferase, allowed *in vitro* imaging of necrosis. We further confirmed real-time imaging of intratumoral necrosis caused by physical or chemical tissue disruption, validating its application in *in vivo* necrosis imaging. Thus, the constructed imaging system could be a powerful tool for the optimization of the therapeutic condition for cancer therapy and for the evaluation of novel anticancer drugs targeting necrosis.

INTRODUCTION

Necrosis is a form of cell death that is biochemically uncontrolled and caused by internal or external stresses, such as mechanical stimuli, chemical agents, or pathogens, unlike apoptosis, which involves a highly regulated and elaborate network of biochemical events and cascades[1,2] (although necroptosis, a controlled necrosis-like cell death through a signaling network, has now been reported[1,3–5]). The process leading to necrosis is usually rapid, causing cell swelling, dilation of mitochondria and the endoplasmic reticulum, and cell membrane rupture due to dysregulation of ion homeostasis or osmodysregulation.[1,2,6] An influx of calcium ions from the extracellular matrix activates intracellular calpains, which contribute to the degradation of membrane, cytoplasmic, and nuclear substrates, leading to the breakdown of cellular architectures.[2] In addition, freed lysosomal hydrolases, including cathepsin B and D, degrade nucleic acids and proteins. In particular, cathepsin D—unlike some other cathepsins that are activated at low pH—cleaves various substrates, such as fibronectin and laminin at near-neutral pH. Eventually, cell lysates released into the extracellular compartment activate leukocytes, lymphocytes, and macrophages, which phagocytose the remnants of dead cells, triggering inflammatory responses.[7] Thus, necrosis can also become a common indicator of the development of various diseases.

In general, protein splicing (PS) is a post-translational process catalyzed by an intervening polypeptide unit, called intein, between another type of polypeptide unit, called extein, which is present at both termini, in the same polypeptide molecule.[8] In this process, the intein facilitates a reaction to unite the two exteins to become a shorter polypeptide molecule. Among various types of PS, one called protein *trans*-splicing (PTS) involves a different type of intein divided into two parts,[9] the N-terminal and the C-terminal splicing domains (DnaEn and DnaEc), which reside at the N terminal and C terminal of two different polypeptide molecules. These molecules are composed of two different units called exteins. When these two molecules are associated in close vicinity, ligation of the exteins of these two molecules is catalyzed through refolding between DnaEn and DnaEc.[10,11] For the PS reactions, exogenous cofactors or energy sources, such as ATP or GTP, are not required.

Received 16 September 2020; accepted 5 December 2020;
https://doi.org/10.1016/j.omto.2020.12.001.

Correspondence: Ryohei Ogawa, PhD, Department of Radiological Sciences, Graduate School of Medicine and Pharmaceutical Sciences, University of Toyama, Toyama 930-0194, Japan.
E-mail: ogawa@med.u-toyama.ac.jp

 © 2020 The Author(s).
This is an open access article under the CC BY license (http://creativecommons.org/licenses/by/4.0/).

Since its discovery, PTS has been harnessed for the development of several protein-engineering methods or the creation of artificial enzymes with new functions.[12,13] Applying a PTS reaction, Kanno et al.[14] successfully constructed a real-time imaging system to detect apoptosis in xenograft tumors in nude mice. They utilized a cyclic luciferase (luc) system in which the two split-luc gene fragments were connected in inverse order with a DNA fragment coding for the DEVD peptide sequence, which can be cleaved by caspase-3 (cas-3), in addition to DNA fragments coding for a DnaE intein placed at the 5′ and 3′ (both) ends of the reconnected luc gene. A polypeptide translated from this entire DNA fragment is circularized with the PTS activity of the DnaE. Although the luc circularized by the ligation of both terminals loses its enzymatic activity, the linearized luc formed as a result of cleavage at the DEVD motif during the apoptotic process was restored to the wild-type structure and regained its enzymatic activity, enabling light emission by oxidation of luciferin. Furthermore, we devised this system to only work in a hypoxic environment so that it can be utilized for screening anticancer drugs targeting cancer stem cells, since they tend to reside in hypoxic regions.[15]

In radiation therapy, a major component of cancer therapy (along with surgery and chemotherapy), the frequency at which apoptosis is induced in tumors is known to be as low as several percent, regardless of the p53 status.[16] In addition, radiation not only directly causes necrosis in tumor cells but also causes tumor vascular damage, resulting in secondary necrosis in tumor cells via oxygen and nutrient depletion.[17] Thus, the main form of cell death induced in tumors after radiation therapy is thought to be necrosis, not apoptosis.[6,16] On the other hand, many anticancer drugs used in chemotherapy also cause cell death by necrosis as well as apoptosis.[18] After treatment with cisplatin, which is widely used in the treatment of a variety of cancers, both apoptosis and necrosis are induced in tubular epithelial cells in the proximal tubule, resulting in tubular damage in approximately one-third of treated patients.[19–23] For these reasons, the optimization of irradiation conditions in radiation therapy and the clinical doses used in chemotherapy should be examined with the evaluation of necrosis as a therapeutic effect index. The development of a highly sensitive system to detect necrosis via effective imaging techniques is therefore urgently required. Regarding an *in vivo* imaging system for necrosis, Fang et al.[24] successfully detected necrotic tissue for real-time surgery using indocyanine green (ICG), a US Food and Drug Administration (FDA)-approved near infrared (NIR) fluorescent dye, in different organs of different animal models. However, in general, the change in the fluorescence intensity signal is very small, and most of the lights for excitation and emission are absorbed while passing through living tissues, making it difficult to apply in the clinical setting.

To address this issue, we constructed a minimally invasive, real-time imaging system to visualize intratumoral necrosis by bioluminescence. In this report, we describe the construction of our novel necrosis imaging system and show the detection and quantification of necrosis that was chemically and physically induced in tumors. This system could therefore play a unique role in determining the best therapeutic condition for radiation therapy and other cancer therapies.

RESULTS

In vitro functional validation of necrosis imaging reporter

Necrosis, unlike apoptosis, is characterized by the release of cell contents. Thus, when necrosis occurs in cells expressing either of two split-luc fragments, due to cell membrane destruction, the two fragments released from cells meet to be reconstituted in the extracellular space by PTS. The addition of luciferin as a luminescent substrate to the extracellular space where luc is reconstituted is presumed to result in the generation of bioluminescence, enabling imaging of necrosis (Figure 1). To evaluate whether or not this methodology actually works as an *in vitro* and *in vivo* experimental system, we constructed plasmid vectors to express a pair of necrosis imaging reporters, pFLucN and pFLucC, in which a DnaE intein sequence involved in PTS was linked to either of two split-firefly luc (FLuc) fragments. In addition, we constructed two plasmid vectors (pNLucN and pNLucC) that express another pair of two split-luc fragments from a deep sea shrimp luc (NLuc), which produces more intense bioluminescence and shows higher thermal stability in comparison to FLuc. Furthermore, another pair of plasmid vectors of pNLucN/v2 and pNLucC/v2, which are identical to the latter pair, except for the hinge sequence mediating between the luc derivative fragments and the DnaE inteins, was also constructed (see Materials and methods and Figure 2). After Chang liver cells were transiently transfected with these plasmids, necrosis was induced with passive lysis buffer (PLB), a surfactant. When D-luciferin was added to a lysate from cells transfected with pFLucN or pFLucC expressing either of the necrosis imaging reporters (FLucN or FLucC), no bioluminescence was detected (equivalent to the background). However, a significant increase in the bioluminescence value was observed in comparison to background when equal volumes of both cell lysates were mixed. In the case of cells expressing either of the necrosis imaging reporters of NLuc without the hinge sequence (NLucN/v2 or NLucC/v2), when both cell lysates were mixed, the bioluminescence value increased approximately five-fold in comparison to that in the case of FLuc reconstitution (Figure 3A). These results suggest that when released extracellularly due to necrosis, either pair of necrosis imaging reporters could be reconstituted for enzyme activity via PTS, enabling the detection of necrosis. For high-sensitivity visualization of the necrosis induced in tumors, a reconstituted luc whose enzymatic activity is maintained for a long time, even at mouse body temperature, is desired. Thus, we compared the thermal stability of enzymatic activities at 37°C with two reconstituted lucs, FLuc and NLuc, employing two pairs of cell lines that stably express a pair of necrosis imaging reporters for FLuc (FLucN and FLucC) or NLuc (NLucN/v2 or NLucC/v2). The maximum bioluminescence value generated with the reconstituted FLuc was obtained immediately after mixing the lysates of the cell pair, and it rapidly decreased to approximately 1/10 in 30 min. For the reconstituted NLuc/v2 without the hinge sequence, on the other hand, the relative bioluminescence value was approximately 100 times higher in comparison to FLuc and was not attenuated, even after 3 h at 37°C (Figure 3B). Based on these results, we decided to

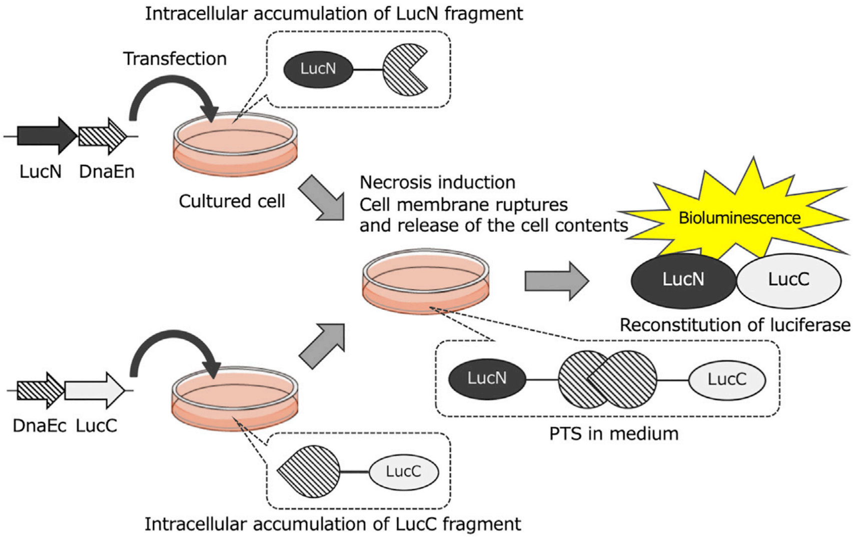

Figure 1. Schematic illustration of the principle of our minimally invasive real-time imaging system for necrosis

This system is based on the reconstitution of two split-luc fragments (LucN and LucC) mediated with DnaE intein sequences that were linked to the fragments, which catalyzes PTS for the reconstitution. Once necrosis is induced on a cell mixture expressing either one of the necrosis imaging reporters, the cell's constituents that include them are spilled into the surroundings through the plasma membrane when it is ruptured by necrosis. This enables the interaction of the necrosis imaging reporters and subsequently luc reconstitution as a result of PTS. Thus, the luc enzyme is then ready to generate bioluminescence.

employ NLucN/v2 and NLucC/v2 as the necrosis imaging reporter for further experiments.

Improvement of the necrosis imaging reporter for more intense bioluminescence signaling

Under conditions where necrosis is sporadically induced for a long period in the tumor, the split-NLuc fragments that are released in the extracellular space are expected to be degraded by intracellular and extracellular proteases during the acquisition of enzymatic activity by reconstitution, leading to a decrease in the bioluminescence signal. To detect necrosis even under such conditions, we attempted to construct improved necrosis imaging reporters for stronger bioluminescence signals. NLuc, which consists of 171 amino acid residues, was divided into two fragments, the N-terminal fragment of 160 amino acids in length (NLucN) and the C-terminal fragment of 11 amino acids in length (NLucC). By adding the sequences involved in protein stabilization to these two fragments, we aimed to improve the necrosis imaging reporters, by improving their resistance to degradation by proteases. For stabilization of the N-terminal fragment, NLucN/v2-Fc was newly constructed by fusing a DNA fragment coding for the Fc region of human immunoglobulin G1 (hIgG1-Fc), which is involved in protein stabilization to the C-terminal of the NLucN/v2 fragment. Lysates from cells transfected with a plasmid (pNLucN/v2 or pNLucN/v2-Fc) were treated at 37°C for up to 60 min. Each of them was then mixed with the same amount of lysate from cells transfected with the pNLucC/v2 plasmid for the evaluation of the thermal stability of these N-terminal fragments based on their bioluminescence values with NLucC/v2. As shown in Figure 4A, the relative bioluminescence value by the NLucN/v2-Fc fragment was approximately twice as intense as that by the NLucN/v2 fragment without incubation at 37°C. However, the bioluminescence values of the NLucN/v2 and NLucN/v2-Fc fragments with NLucC/v2 treated at 37°C for 60 min were decreased by approximately 1/5 and 1/100, respectively, in comparison to the values obtained without the 37°C treatment, suggesting that the NLucN/v2 fragment is more stable outside of cells than the NLucN/v2-Fc fragment. Since the C-terminal fragment is composed of only 11 amino acid residues (its N-terminal counterpart is composed of 160 residues), it would have a greater effect on the generation of the bioluminescence than its N-terminal counterpart if the either fragment is similarly degraded in the extracellular environment. We therefore improved the C-terminal fragment to increase its thermal stability; NLucC/v2-Fc was obtained by fusing a fragment containing an hIgG1-Fc to the C-terminal of the NLucC/v2 fragment, NLucC/v2-FLuc was created by fusing FLuc with thermal stability to the C-terminal of the NLucC/v2 fragment, and NLucC/v2-Fc/FLuc was generated by fusing both hIgG1-Fc and FLuc to the N and C terminals of the NLucC/v2 fragment, respectively. The thermal stability of these constructed C-terminal fragments was also evaluated based on their bioluminescence values with NLucN/v2 in the same manner as the N-terminal fragment stability. As shown in Figure 4B, when mixed with cell lysate containing NLucN/v2, all of the improved C-terminal fragments showed higher bioluminescence values in comparison to the NLucC/v2 fragment. In particular, the bioluminescence value generated by NLucC/v2-Fc was approximately 100 times higher than that generated by NLucC/v2 without thermal treatment, and its declining curve of bioluminescence during incubation at 37°C was the most gradual, showing the highest thermal stability among all of the improved C-terminal NLuc fragments. It was anticipated that the intensity of the bioluminescence would be dependent upon ratios between the cell contents with NLucN/v2 and NLucC/v2-Fc. To confirm this, changes in the bioluminescence values of cell mixtures consisting of different ratios of the cells were evaluated using cells stably expressing the NLucN/v2 fragment and NLucC/v2-Fc fragment. As a result, it was found that the bioluminescence value increased as the ratio of the cells with NLucC/v2-Fc increased; however, the increase was saturated when the ratio between cells with NLucN/v2 and NLucC/v2-Fc reached 1:10 (Figure 4C, bottom panel). Similar results were obtained in the visualized images (Figure 4C, top panel). To determine the best cell combinations for bioluminescence signals, we evaluated various combinations of cells with an N-terminal fragment and its C-terminal fragment counterpart. Before the bioluminescence values were measured, each cell lysate was independently

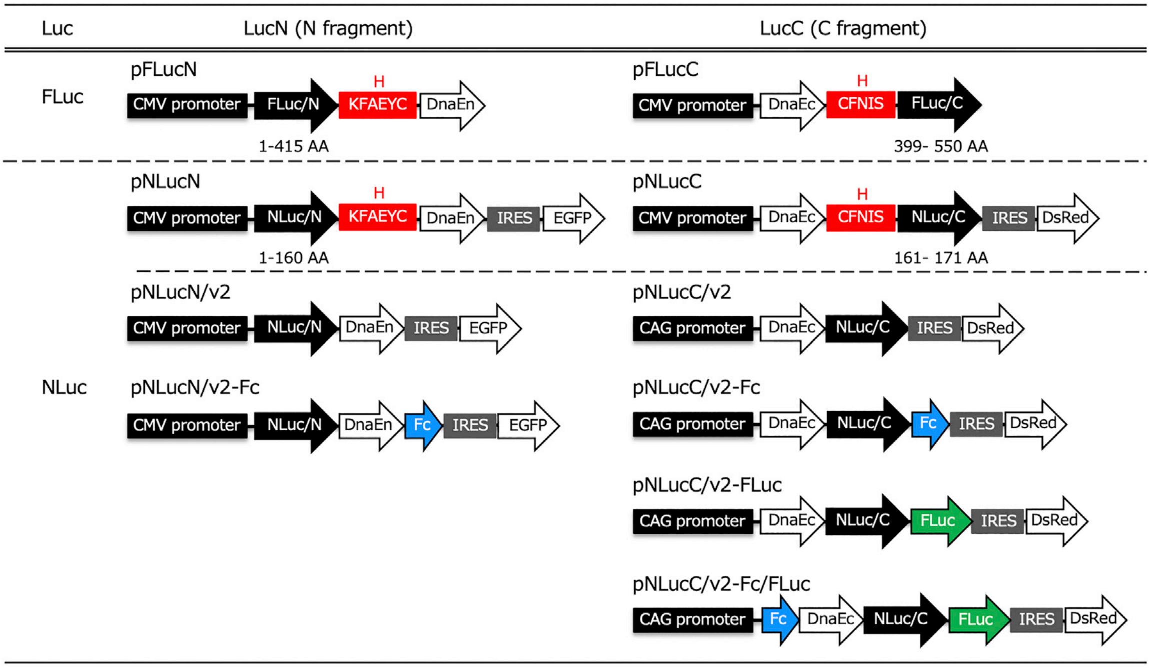

Figure 2. Schematic diagram for the domain structures of necrosis imaging reporters

Reporters were constructed by connecting a split-luc fragment (N fragment or C fragment) to a DnaE sequence and other sequences. All domains were depicted as rectangles or arrows. FLuc, firefly luciferase; NLuc, deep sea shrimp luciferase; H, hinge sequence (one-letter amino acid code); IRES, internal ribosome entry site; Fc, human IgG1-Fc region; DnaEn, N-terminal fragment of DnaE intein; DnaEc, C-terminal fragment of DnaE intein.

incubated at 37°C for up to 120 min and then mixed at a ratio of 1:1 or 1:10 (cells with N-terminal fragment: cells with C-terminal fragment) to evaluate the combination of cells with the highest bioluminescence values. In the cases of the combinations of FLucN and FLucC or NLucN/v2 and NLucC/v2, their bioluminescence values rapidly declined by about 1/10 or less to compare with that without 37°C treatment in 60 min. In contrast, the combination of cells with NLucN/v2 and NLucC/v2-Fc showed the best bioluminescence signals, and the activity of the reconstituted luc showed the best thermal stability after they were mixed at the ratio of 1:10. The bioluminescent value obtained with the combination of cells with NLucN/v2 and NLucC/v2-Fc was 50,000 times higher than that obtained by the combination of cells with FLucN and FLucC, confirming that the combination of NLucN/v2 and NLucC/v2-Fc was the best necrosis imaging reporter (Figure 4D). Next, to demonstrate the wide applicability of this imaging system, we used two more cell lines, MCF-7 and A549, derived from different organs. Each cell line was transiently transfected with pNLucN/v2 or pNLucC/v2-Fc, and the two transfectants were combined and subjected to bioluminescence measurement. As shown in Figure S1, the combination of MCF-7 transfectants and the combination of A549 transfectants showed bioluminescence values that were 0.4 and 0.03 times that obtained with Chang liver cells, respectively. While these values were not very intense, both were significantly higher than the value derived from either of the two single transfectants, indicating that this imaging system could work in a broad range of tissues.

Detection of necrotic cells with a flow cytometer and fluorescence microscope and bioluminescent imaging (BLI) of *in vitro* cellular necrosis

To evaluate whether the PLB could actually induce necrosis in Chang liver cells, flow cytometric measurements were carried out after PLB-treated cells were stained with fluorescein isothiocyanate (FITC) Annexin V and Ethidium Homodimer III (EthD-III). The percentage of the cells stained with EthD-III and both probes after the PLB treatment clearly increased up to 16-fold in comparison to that before the treatment, confirming that PLB induced necrosis (Figure 5A). Next, to further confirm that the contents of cells, including protein, could leak out of cells upon the induction of necrosis by PLB, stably transfected cell lines with pNLucN/v2 and pNLucC/v2-Fc were used, since these cell lines produce enhanced green fluorescent protein (EGFP) and red fluorescent protein (Ds-Red), respectively (see Figure 2). These cell lines were observed under a fluorescence microscope before and after treatment with PLB. Both colors observed in the cells disappeared after these cells were treated with the PLB, suggesting that most of the contents, including two split-luc fragments, together with the fluorescence proteins that resided inside the cells, could leak into the extracellular space due to necrosis (Figure 5B). From these two experiments, it was confirmed that PLB treatment could surely induce necrosis in cells and that, upon necrosis, contents that probably included necrosis imaging reporters could be released from the cells. We then investigated whether the chosen combination of necrosis imaging reporters, NLucN/v2 and NLucC/v2-Fc, could be

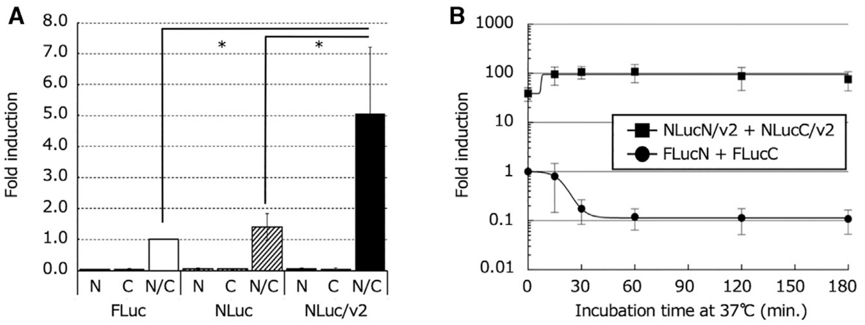

Figure 3. *In vitro* functional validation of necrosis imaging reporters

(A) Quantitative analysis of the bioluminescence intensity according to the interaction of necrosis imaging reporters. Chang liver cells transiently transfected with a plasmid (pFLucN, pFLucC, pNLucN, pNLucC, pNLucN/v2, or pNLucC/v2) were recovered at 12 h after transfection. Recovered cells were lysed solely or pairwise in PLB and then incubated at an ambient temperature before the luc assay. Their bioluminescence values were normalized to that of the cell lysate from the mixture of FLucN-transfectants and FLucC-transfectants (FLucN/C). They were plotted as the mean fold induction. Error bars represent the standard deviation (n = 5–7). Asterisks indicate statistical significance ($p < 0.01$). (B) Thermal stability of enzymatic activity (bioluminescence generation) of the reconstituted luc. A combination of FLucN-expressing cells and FLucC-expressing cells, or NLucN-expressing cells and NLucC-expressing cells, was lysed in PLB and then incubated at 37°C for the indicated durations before the luc assay. Their bioluminescence values were normalized to that of the cell lysate from the mixture of FLucN-expressing cells and FLucC-expressing cells without incubation (t = 0 min) and plotted as the mean fold induction. The error bars represent the standard deviation.

released upon necrosis and extracellularly assembled for the visualization of bioluminescence. A cell line expressing NLucN/v2 or NLucC/v2-Fc was treated with the PLB, NP-40, or ultrasonication to induce necrosis and then individually incubated at 37°C for 60 min. The cell lysates were mixed equally or at a ratio of 1:10 on a dish and then bioluminescence images were captured using an electron-multiplying charge-coupled device (EMCCD) camera. As shown in Figure 5C, we successfully captured images generated due to *in vitro* cellular necrosis. The highest-intensity image was obtained by a 1:10 combination of cell lysates of cell lines expressing NLucN/v2 or NLucC/v2-Fc, respectively, prepared with the PLB, NP-40, or ultrasonication.

BLI of necrosis induced tumors in an animal model

Solid tumors often form necrotic regions due to an imbalance between cell proliferation and angiogenesis. To confirm whether or not regions of necrosis were actually formed in xenograft tumors, a tumor that was formed by a transplanted 1:10 cell mixture stably expressing NLucN/v2 and NLucC/v2-Fc, respectively, was excised. No significant difference was observed in the proliferation rate of the cells stably expressing NLucN/v2 or NLucC/v2-Fc, respectively, *in vitro* (Figure S2). As shown in the Materials and methods, the tumor tissue was processed to prepare thin sliced sections, and then the sections were subjected to hematoxylin and eosin (H&E) staining. As shown in Figure 6A, necrotic regions were obvious, with a loss of cellular detail and with cell debris present in the tissue section, confirming that necrosis was formed during tumor growth inside the xenograft tumor. Next, to confirm whether intratumoral necrosis formed by tumor growth can actually be visualized, we administered 100 μL of furimazine solution (50× dilution in sterile PBS) to obtain BLI of necrosis in tumors in the animal model. Although no bioluminescence signal from xenograft tumors was detected when mice were injected intraperitoneally or intravenously with the substrate, strong bioluminescence signals were obtained when it was intratumorally injected (Figure 6B), enabling the visualization of intratumoral necrosis formed during tumor growth. Finally, we attempted to visualize the *in vivo* necrosis induced by physical or chemical destruction. Necrosis was induced with needlestick injuries or CelLytic MT mammalian tissue lysis/extraction reagent treatment, as physical or chemical disruption, respectively. The bioluminescence values from tumors after needlestick picking and tissue lysis reagent injection were significantly increased 3.3-fold and 4.6-fold, respectively, in comparison to untreated tumors (Figure 7B). In this study, we established a minimally invasive system for real-time BLI of tumor necrosis using NLucN/v2 and NLucC/v2-Fc as a reporter (Figure 7A).

DISCUSSION

In this study, we demonstrated BLI of *in vitro* cellular necrosis with a novel necrosis imaging system consisting of two types of necrosis imaging reporters. The principle of this imaging system is based on reconstitution of necrosis imaging reporters, in which a DnaE intein sequence was linked to the two split-luc fragments. When necrosis occurred in a tumor composed of a cell mixture harboring either of the necrosis imaging reporters, due to cell membrane destruction, the two luc fragments released from the cells met to be reconstituted in the extracellular space by PTS. The reconstituted luc could resume the emission of bioluminescence upon the addition of luciferin, enabling imaging of necrosis (Figure 1). In fact, the necrosis imaging reporters constructed by dividing FLuc or NLuc into two fragments were reconstituted by PTS, restoring the luc activity (Figures 3A and 3B). However, with regard to the protein stability of luc after reconstitution, the stability of NLuc was much higher than that of FLuc and was not attenuated, even after 3 h of incubation at 37°C (Figures 3A and 3B). This may be because the intact NLuc originally shows higher thermal stability, retaining its activity even after incubation at 55°C for 30 min,[25] while the intact FLuc begins to lose activity, even at temperatures below 30°C. During the induction of necrosis, the NLucN or NLucC fragments that are released in the extracellular space are expected to be degraded by intracellular and extracellular proteases before acquiring enzymatic activity by reconstitution, leading to decreased bioluminescence signaling. Of course, the bioluminescence signal of the reconstituted NLuc is inferior to its intact counterpart since the enzyme reconstitution is not perfect in its activity or stability.

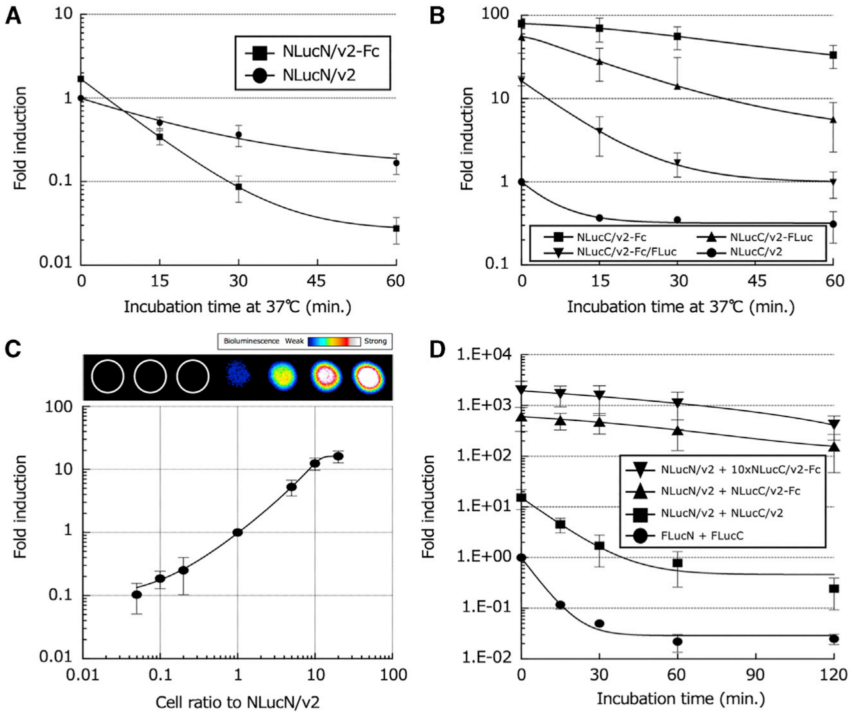

Figure 4. Quantitative bioluminescence analyses generated with the improved necrosis imaging reporters with stronger bioluminescence signals

(A) Thermal stability of the improved necrosis imaging reporters containing NLucN including NLucN/v2 and NLucN/v2-Fc in cell lysate. After transient transfection with a plasmid (pNLucN/v2 or pNLucN/v2-Fc), cells were lysed in PLB and incubated at 37°C for the indicated duration. Bioluminescence measurements were carried out by mixing each lysate with similarly prepared lysate of cells stably expressing NLucC/v2 without incubation at 37°C. The obtained bioluminescence intensities were normalized against that obtained from a lysate mixture with NLucN/v2 and NLucC/v2 without incubation at 37°C. The data were plotted as the mean fold induction. The error bars represent the standard deviation. (B) Thermal stabilities of the improved necrosis imaging reporters containing NLucC, including NLucC/v2, NLucC/v2-Fc, NLucC/v2-FLuc, and NLucC/v2-Fc/FLuc in cell lysates. After transient transfection with a plasmid (pNLucC/v2, pNLucC/v2-Fc, pNLucC/v2-FLuc, or pNLucC/v2-Fc/FLuc), cells were lysed in PLB and incubated at 37°C for the indicated duration. Bioluminescence measurements were carried out by mixing each lysate with similarly prepared lysate of cells stably expressing NLucN/v2 without incubation at 37°C. The obtained bioluminescence values were normalized to that obtained from lysate mixture with NLucN/v2 and NLucC/v2 without incubation at 37°C. The data were plotted as the mean fold induction. The error bars represent the standard deviation. (C) Bioluminescence intensity enhancement by adjusting mixing ratios of cells expressing NLucN/v2 or NLucC/v2-Fc. Different ratios of cells expressing NLucN/v2 or NLucC/v2-Fc were lysed in PLB and subjected to bioluminescence measurement. Image on the top of the graph represents a BLI obtained from lysates of cell mixture at different ratios. The data were plotted as the mean fold induction. The error bars represent the standard deviation. (D) Thermal stabilities of variable necrosis imaging reporters in cell lysate. Cells expressing a necrosis imaging reporter were independently lysed in PLB and then incubated at 37°C for the indicated duration. Bioluminescence measurements were carried out by mixing a pair of the incubated lysates containing NLucN and NLucC. The obtained bioluminescence values were normalized to the value obtained from a mixture of lysate with FLucN and FLucC without incubation at 37°C. The data were plotted as the mean fold induction. The error bars represent the standard deviation.

To detect necrosis under such conditions, we attempted to construct an improved necrosis imaging reporter with a stronger bioluminescence signal. One of the necrosis imaging reporters was successfully improved by strengthening the bioluminescent signal with the addition of sequences that improve protein stability, such as hIgG1-Fc and/or FLuc to the NLucC/v2 fragment (Figure 4B). However, the same addition to the NLucN/v2 fragment did not change the resultant bioluminescence (Figure 4A). This may be due to the stability of either fragment, since the sizes of the fragments were so different (NLucN/v2 is about 10 times larger than NLucC/v2, which is composed of 11 amino acids). Therefore, NLucN/v2 could itself fold a sufficiently stable structure without the addition of sequences that improve protein stability. Interestingly, in these cases for improvements by adding protein fragments, two different types of protein stability were observed with the NLucC/v2 fragment. As observed when Fc, FLuc, or both were added, the stability of NLucC/v2 significantly increased inside cells, since the bioluminescence observed was greater than that observed with the intact NLucC/v2 if they were not incubated at 37°C, as shown in Figure 4B. This may be due to the facilitated accumulation of the NLucC/v2 fragment inside the cells. The other was stability outside the cells, which was only observed when Fc was added to NLucC/v2, since only NLucC/v2 with the addition of Fc showed a slower bioluminescence diminution rate, which would be due to higher protein stability in comparison to intact NLucC/v2 (Figures 4B and 4D). In addition, we found that the bioluminescence value depended on the mixing ratio between cells expressing NLucN/v2 and NLucC/v2-Fc fragments and reached the maximum at the mixing ratio of 1:10 or more (Figure 4C). When the cells were mixed at the ratio and necrosis was induced using PLB, NP-40, or ultrasonication, we successfully captured images of *in vitro* cellular necrosis. Furthermore, we demonstrated that this imaging system could be applied to A549 and MCF-7 cell lines, indicating its utility with cells of different organ origins from the Chang liver cell line. This may indicate the wide applicability of this system, although the bioluminescence values obtained after necrosis induction were relatively low, possibly due to the low number of necrosis imaging reporters available, which may be attributed to differences in transfection efficiency and/or promoter activity. The above results showed that the necrosis imaging system that we designed and constructed was useful for capturing images of cellular necrosis *in vitro* (Figure 5C).

We also demonstrated real-time BLI of intratumoral necrosis formed during tumor growth or induced with needlestick injuries or surfactant, as a physical or a chemical disruption, respectively (Figures 6B and 7A). Although no bioluminescence signal from xenograft tumors was

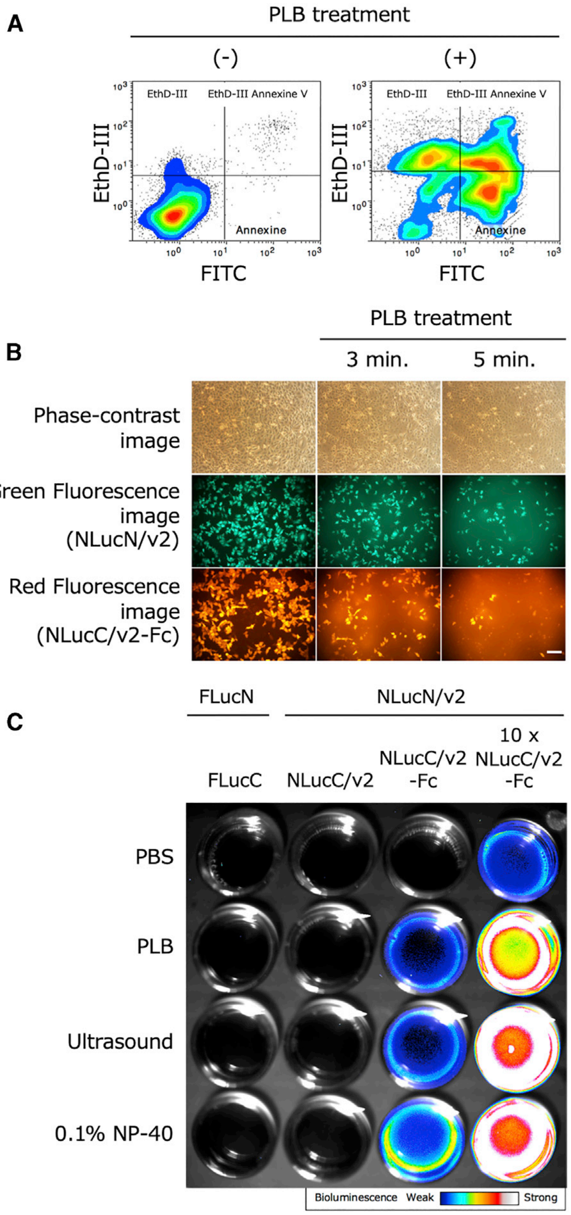

Figure 5. Detection of necrotic cells with a flow cytometer and fluorescence microscope and BLI of *in vitro* cellular necrosis

(A) Representative flow cytometry dot plots with double Annexin V-FITC/EthD-III staining for Chang liver cells treated with PLB for 5 min. (B) Cells stably expressing NLucN/v2 or NLucC/v2-Fc, which also express GFP or Ds-Red, respectively, were treated with PLB. Fluorescence microscopy images were acquired at 3 and 5 min after PLB treatment. Scale bar, 100 μm. (C) 5.0×10^5 cells stably expressing necrosis imaging reporters (FLucN, FLucC, NLucN/v2, NLucC/v2, or NLucC/v2-Fc) were treated with PLB, ultrasound, 0.1% NP-40, or PBS (control) and then incubated independently at 37°C for 60 min. BLI was performed for 5 min after mixing equal volumes of two cell lysates with 500 μM D-luciferin for the FLucN and FLucC combination or 50-fold diluted furimazine for the other combinations using an EMCCD camera.

detected when mice were injected intraperitoneally or intravenously with furimazine, strong bioluminescence signals were obtained when it was injected intratumorally. It has been reported that furimazine is more unstable in the body than D-luciferin, and, in circulation, the furimazine concentration rapidly dropped by 99.7% in 20 min after administration to mice, suggesting that the rapid elimination of furimazine from circulation is due to its consumption by the reticuloendothelial system.[26] Thus, it is considered that the bioluminescence signal was not obtained by intravenous (i.v.) or intraperitoneal (i.p.) administration and that it was only detected after intratumoral administration.

The salient feature of the present method is that the necrosis induced in the tumor can be visualized by bioluminescence in real time with minimal invasiveness. To date, *in vivo* BLI of necrosis was reported by Fang et al.[24] using ICG that emits fluorescence. However, the major limitation of this technique is the shallow imaging depth due to its fluorescence, and they pointed out that this problem might lead to false-negative detection of the necrotic lesions deep inside organs.[24] On the other hand, our system, which utilizes bioluminescence to visualize necrosis, has a higher signal-to-noise ratio in comparison to fluorescence and enables deep observation in the living body without an excitation light. However, the problem of the present system is that the substrate furimazine is unstable *in vivo*, resulting in low signal intensity from intratumoral necrosis. Kuchimaru et al.[27] have developed a novel luciferin analog, AkaLumine-HCl, which produces a bioluminescence with 675 nm (near-infrared wavelength) and greatly improved the light transmission efficiency of tissue. AkaLumine-HCl bioluminescence displays 5- or 8.3-fold higher light transmission than D-luciferin bioluminescence in 4- or 8-mm-thick tissue sections, respectively.[27] In addition, Iwano et al.[28] have successfully developed Akaluciferase, an improved FLuc that is enzymatically optimal for AkaLumine-HCl and that exhibits higher thermal stability than FLuc; the combination with AkaLumine-HCl produces bioluminescence with 20 times the intensity of the bioluminescence produced by the conventional combination of D-luciferin and FLuc.[28] The problem of low bioluminescence from intratumoral necrosis might be solved by the reconstruction of our necrosis imaging system, taking advantage of the newly developed luciferase-substrate combination.

Finally, we have developed a promising BLI system. This could be applied to develop a system to more precisely and quickly evaluate the reactions of tumors to radiation therapy or chemotherapy in comparison to conventional methods of evaluation. This system should be very useful for the evaluation of novel therapies for cancer or necrosis-associated diseases, facilitating the development of novel therapeutic modalities. Currently, although there are still some defects, we would like to complete this system, which works in a dynamic and minimally invasive manner.

MATERIALS AND METHODS

Cell culture and bacteria

Chang liver cells (HeLa contaminant), A549 human lung adenocarcinoma cells, and MCF-7 human mammary carcinoma cells were grown in RPMI 1640 medium supplemented with 10% (v/v) heat-inactivated

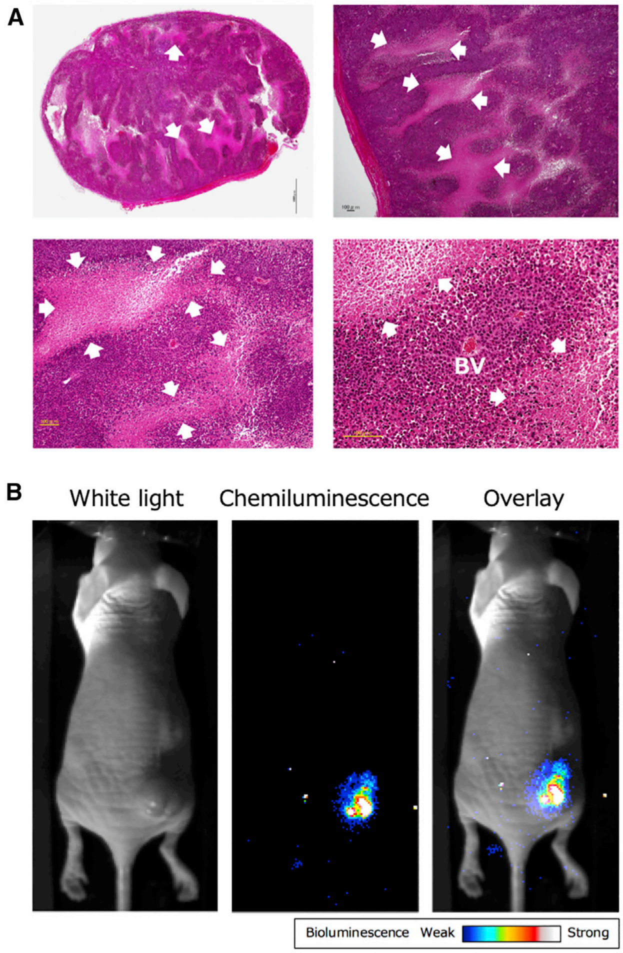

Figure 6. *In vivo* BLI of necrosis in implanted tumors composed of cells stably expressing the necrosis imaging reporters, NLucN/v2 and NLucC/v2-Fc

(A) H&E-stained tissue section of an implanted tumor with necrosis. White arrows indicate the areas of necrosis. BV, blood vessel. (B) Representative BLI of necrosis caused by tumor growth in a xenograft tumor (8–10 mm in maximum diameter). This image was acquired using an EMCCD camera after the direct intratumoral injection of 100 μL of 50-fold diluted furimazine solution.

fetal calf serum, 100 U/mL penicillin, and 100 μg/mL streptomycin in a 5% CO_2 incubator at 37°C. All cell lines were purchased from ATCC (Rockville, MD, USA). The DH5α strain of *Escherichia coli* (Takara Bio, Ohtsu, Japan) was used for the DNA manipulation experiments. The *E. coli* cells were grown in Luria-Bertani (LB) medium at 37°C. All medium compositions for *E. coli* were purchased from BD Diagnostics (Sparks, MD, USA).

Plasmid construction

We constructed plasmid vectors for a necrosis imaging reporter in which a DnaE intein sequence (DnaEn or DnaEc) involved in PTS was linked to either of two divided luc fragments. Schematic representations of the structural features of the plasmids used in this study are depicted in Figure 2. For a FLuc reconstitution system, two plasmids were constructed. One was pFLucN, which was constructed by cloning a DNA fragment covering a 5′-terminal sequence of the FLuc gene (1-1245 nt), a so-called hinge sequence (KFAEYC in one-letter amino acid code), and DnaEn in the BamHI site, which was introduced immediately downstream of the cytomegalovirus (CMV) promoter in a plasmid, pCMV15. The other was pFLucC, which was constructed by cloning a DNA fragment covering DnaEc, another hinge sequence (CFNIS), and a 3′-terminal fragment of the FLuc gene (1198-1650 nt) in the BamHI site of pBApo-CMV Neo (Takara Bio). A few different combinations of plasmids were constructed for NLuc reconstitution. Using pCMV-cNLuc, which was constructed by replacing the FLuc gene of pCMV with the NLuc gene as a template, NLucN, a DNA fragment covering a 5′-terminal fragment of the NLuc gene (1-480 nt), a hinge sequence (KFAEYC), and DnaEn, was PCR-amplified with tag sequences of the BamHI site or EcoRI site added to the 5′ or 3′ ends, respectively. This fragment was inserted into pIRES2-AcGFP1 (Takara Bio) to construct pNLucN. Likewise, using pCMV-cNLuc as a template, NLucC, a DNA fragment covering DnaEc, a hinge sequence (CFNIS), and a 3′-terminal fragment of NLuc gene (484-513 nt) was PCR-amplified with tag sequences of the BamHI site or EcoRI site added to the 5′ or 3′ ends, respectively. This fragment was inserted into pIRES2-DsRed-Express2 (Takara Bio) to construct pNLucC. Using the pNLucN plasmid as a template, inverse PCR was conducted to exclude the hinge sequence. The amplified sequence was self-ligated after digestion with MluI to construct pNLucN/v2. Likewise, using the pNLucC plasmid as a template, inverse PCR was conducted to exclude the hinge sequence. The amplified sequence was self-ligated after digestion with MluI, and the CMV promoter was replaced with the CAG promoter to construct pNLucC/v2. We then constructed pNLucN/v2-Fc and pNLucC/v2-Fc. The former is a plasmid in which a hIgG1-Fc was connected to the 3′ end of NLucN/v2, expressing the fusion protein. The latter is a similar plasmid, in which the same hIgG1-Fc DNA fragment was connected to the 3′ end of NLucC, which expressed the fusion protein. These plasmids were constructed by introducing a PCR-amplified DNA fragment containing the hIgG1-Fc region from pCAG-Neo hIgG1-Fc (Fujifilm, Tokyo, Japan) with tags of the BamHI site and the MluI site at both ends into the BamHI and the MulI site in plasmid pNLucN/v2 or pNLucC/v2. A plasmid, pNLucC/v2-FLuc, which expresses a fusion fragment in which the full length of the FLuc gene is linked to downstream of the NLucC/v2 fragment, was constructed by replacing the Fc region in pNLucC/v2-Fc with the PCR-amplified FLuc gene using a template plasmid, pGL4.13 (Promega). A plasmid, pNLucC/v2-Fc/FLuc, in which hIgG1-Fc and FLuc are linked to the N terminal and C terminal of the NLucC fragment, respectively, was constructed by inserting the PCR-amplified hIgG1-Fc into the upstream of the NLucC/v2-FLuc fragment in pNLucC/v2-FLuc. Structures of all the constructed plasmids were confirmed by nucleotide sequencing analyses.

Transfection and establishment of cell lines stably expressing the necrosis imaging reporter

For transient transfection experiments, 3.0 × 10^5 Chang liver cells, A549 cells, and MCF-7 cells were seeded prior to transfection into a

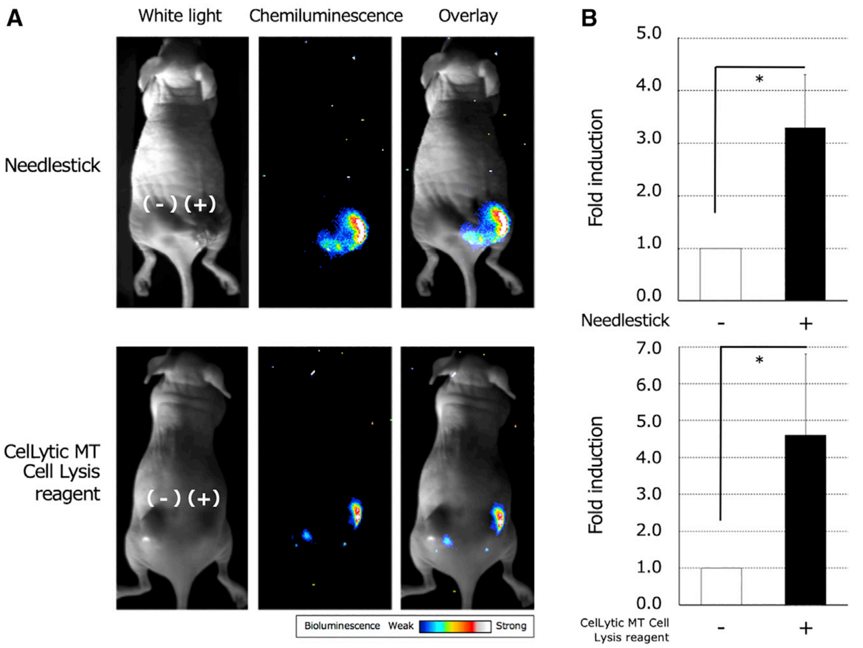

Figure 7. *In vivo* BLI of intratumoral necrosis induced by physical or chemical destruction of xenograft tumors

(A and B) Representative BLI of intratumoral necrosis induced by needlestick or tissue lysis treatment in xenograft tumors (A) and the quantitative analysis of the bioluminescence intensity (B). After needlestick injuries or tissue lysis treatment, *in vivo* BLI of intratumoral necrosis was performed using an EMCCD camera following the direct injection of 20 μL of 50-fold diluted furimazine into tumors (8–10 mm in maximum diameter). The bioluminescence intensity caused by needlestick injuries or tissue lysis treatment was normalized to that of untreated control or vehicle treatment control, respectively. They were plotted as the mean fold induction. Error bars represent the standard deviation (n = 6). Asterisks indicate statistical significance (unpaired t test, $p < 0.05$).

60-mm Petri dish and incubated for 12 h at 37°C. Transfection was performed with the Effectene transfection reagent (QIAGEN, Valencia, CA, USA) in accordance with the manufacturer's instructions.

For the establishment of stably transfected cell lines, 1.0×10^6 Chang liver cells were seeded into a 100-mm culture dish and then transfected with one of the plasmids listed in Figure 2. All the plasmids harbored the neomycin/kanamycin resistance gene, and most of the plasmids harbored the EGFP gene or the Ds-Red gene (no fluorescent protein gene for plasmids pFLucN and pFLucC; Figure 2), for the selection of stably transfected cells. Transfected cells were cultured for 14 days in culture medium containing 10 μg/mL of G418 (Nacalai Tesque, Kyoto, Japan). Harvested cells from antibiotic-resistant colonies stably expressing the EGFP or DsRed, which were identified with a fluorescence microscope, were isolated by limiting dilution using 96-well plates. Isolated cells were proliferated in medium containing 10 μg/mL of G418 to establish cell lines. We used pFLucN, pFLucC, pNLucN, pNLucC, pNLucN/v2, pNLucC/v2, and pNLucC/v2-Fc to establish the FLucN, FLucC, NLucN, NLucC, NLucN/v2, NLucC/v2, and NLucC/v2-Fc cell lines, respectively.

The reconstituted luc activity measurement

Transiently or stably transfected cells (5.0×10^5 cells) with one of the plasmids listed in Figure 2 were washed in PBS once and lysed with 100 μL of PLB (Promega) and incubated at 37°C. To evaluate the thermal stability of the reconstituted luc activity, a mixture of equal volume of a C fragment and the corresponding N fragment were incubated at 37°C for the indicated durations before the luc assay. To evaluate the thermal stability of each fragment separately, transfected cells with a plasmid were lysed in 100 μL of PBL and then incubated at 37°C for the indicated durations without reconstitution by combining with another cell lysate. Then, an equal volume of the corresponding lysate was added and the mixture was subjected to bioluminescence measurement with the Luciferase Assay System or the Nano-Glo Luciferase Assay System using a luminometer (Promega), in accordance with the manufacturer's instructions.

Cell death measurement by flow cytometry

Cell death measurement was performed with an Apoptotic/Necrotic/Healthy Cells Detection Kit (PromoCell, Heidelberg, Germany) in accordance with the manufacturer's instructions. The binding buffer contained in the kit was added to 1.0×10^6 Chang liver cells, which had been collected by centrifugation, and then treated at room temperature for 5 min after PLB treatment. After collecting the cells by centrifugation, FITC Annexin V and EthD-III were added together with the binding buffer. After maintaining the cell suspension in a dark place for 15 min, the ratio of necrotic cells (positive for EthD-III) with or without PLB treatment was determined by flow cytometry (FACSCalibur, Nippon Becton Dicknson, Tokyo, Japan).

Capturing of BLI of *in vitro* cellular necrosis

By centrifugation, 5.0×10^5 cells of the necrosis imaging reporter cells (FLucN, FLucC, NLucN/v2, and NLucC/v2) were collected (for $10 \times$ NLucC/v2-Fc, 5.0×10^6 cells were collected) and then suspended with 100 μL of PLB, 0.1% NP-40, or PBS (control). In addition, physical destruction by ultrasonication was applied to each of the necrosis imaging reporter cells suspended in PBS using an ultrasonic cell crusher (output 25, 10 s, Ultrasonic Processor VP-5T, Taitec, Saitama, Japan). After these treatments, each cell sample was incubated independently at 37°C for 60 min. BLI of *in vitro* cellular necrosis was acquired using an EMCCD camera (iXon3 888, Andor Technology, Belfast, UK) after mixing an equal volume of a cell sample containing the N-terminal luc fragment and a cell

sample containing the C-terminal luc fragment with 500 μM D-luciferin (Wako, Osaka, Japan) at the final concentration or 50-fold diluted furimazine (Nano-Glo luc assay substrate contained in the assay kit, Promega) in sterile PBS. The Andor Solis image analysis software program (Andor Technology, Belfast, UK) was used for analyses of captured images.

Mouse xenograft tumor model

BALB/cA Jcl-nu/nu immunodeficient female mice were obtained from CLEA Japan (Tokyo, Japan) and housed in a light- and temperature-controlled room with *ad libitum* access to water and food. Stably transfected cell mixture containing NLucN/v2 (4×10^5 cells) and NLucC/v2-Fc (2.0×10^6 cells) suspended in PBS was merged with an equal volume (50–100 μL) of Matrigel Matrix (Corning, NY, USA) and subcutaneously implanted at two sites on the backs of 5- to 7-week-old mice. The experiments were performed 2–3 weeks after implantation, when the tumors had grown to 8–10 mm in the maximum diameter. All of the experimental procedures using mice were approved by the animal experiment committee of Kitasato University (authorization number 18-07-2) and were carried out in accordance with the relevant national and international guidelines.

BLI of *in vivo* necrosis induced in living tumors

For *in vivo* BLI of intratumoral necrosis formed by tumor growth, 100 μL of 50-fold diluted furimazine in sterile PBS was administered directly into the xenograft tumor (8–10 mm in maximum diameter) in the animal model. For *in vivo* BLI of the intratumoral necrosis induced by physical destruction, needlestick injuries were made on the tumors (8–10 mm) on the backs of mice anesthetized with a 2% isoflurane/air mixture using an 18 G needle. The scratch of the tumor skin made by the needle was sealed with a skin liquid bandage to prevent leakage of chemiluminescent substrate from the tumor. Then 20 μL of 50-fold diluted furimazine was injected directly into the xenograft tumors with or without needlestick injuries. For *in vivo* BLI of necrosis induced by chemical destruction, CelLytic MT mammalian tissue lysis/extraction reagent (CelLytic MT; Sigma-Aldrich, St. Louis, MO, USA) was injected at a volume of 20 μL into the tumor (8–10 mm). The same volumes of the PBS were given to the control tumor. BLI of *in vivo* necrosis was acquired with an EMCCD camera 10 min after the injection of furimazine. During image acquisition, isoflurane anesthesia was maintained using a nose cone delivery system, and mouse body temperature was controlled with a heat mat. Signal intensity was quantified as the sum of all detected photon counts within a region of interest prescribed over the tumor using the Andor Solis for Imaging software program. Pseudocolor images representing the spatial distribution of the detected photons emitted by luc reconstituted within the tumor were processed using the ImageJ software program (version 1.50i; National Institutes of Health, Bethesda, MD, USA)

Preparation of tumor tissue section

Xenograft tumors excised from mice were fixed with 4% paraformaldehyde for H&E stain. Areas of necrosis in tumor tissue sections were determined by observation of a pathologist.

Statistical analysis

All values are expressed as the mean ± standard deviation. Significant differences between groups were determined by an unpaired t test or a one-way functional ANOVA with Holm-Sidak's multiple comparison test.

SUPPLEMENTAL INFORMATION

Supplemental Information can be found online at https://doi.org/10.1016/j.omto.2020.12.001.

ACKNOWLEDGMENTS

We thank Dr. Ozawa (The University of Tokyo) for sharing the DNA sequence information of a real-time sensing plasmid, pcFLuc-DEVD, for cas-3 activities. This research was supported in part by Grants-in-Aid for Scientific Research (C) (18K07768) from the Japan Society for the Promotion of Science and by grants from Kitasato University School of Allied Health Sciences (Grants-in-Aid for Research Project, nos. 2019-1011, 2018-1012, and 2017-1005).

AUTHOR CONTRIBUTIONS

All authors contributed extensively to the work presented in this paper. G.K. conceived this research concept. G.K., R.O., M.H., and F.H. contributed to the experimental design, data interpretation, and data analysis. A.S. and M.Kato conducted the cell and animal experiments with the help of F. Kojima and F. Kawakami. M.Kubo performed the cell death measurement by flow cytometry. Y.N. and N.A performed the preparation of tumor tissue sections. G.K. and R.O. prepared the manuscript.

DECLARATION OF INTERESTS

The authors declare no competing interests.

REFERENCES

1. D'Arcy, M.S. (2019). Cell death: a review of the major forms of apoptosis, necrosis and autophagy. Cell Biol. Int. *43*, 582–592.
2. Orrenius, S., Gogvadze, V., and Zhivotovsky, B. (2015). Calcium and mitochondria in the regulation of cell death. Biochem. Biophys. Res. Commun. *460*, 72–81.
3. Yuan, J., Amin, P., and Ofengeim, D. (2019). Necroptosis and RIPK1-mediated neuroinflammation in CNS diseases. Nat. Rev. Neurosci. *20*, 19–33.
4. Gong, Y., Fan, Z., Luo, G., Yang, C., Huang, Q., Fan, K., Cheng, H., Jin, K., Ni, Q., Yu, X., and Liu, C. (2019). The role of necroptosis in cancer biology and therapy. Mol. Cancer *18*, 100.
5. Nikoletopoulou, V., Markaki, M., Palikaras, K., and Tavernarakis, N. (2013). Crosstalk between apoptosis, necrosis and autophagy. Biochim. Biophys. Acta *1833*, 3448–3459.
6. Pawlik, T.M., and Keyomarsi, K. (2004). Role of cell cycle in mediating sensitivity to radiotherapy. Int. J. Radiat. Oncol. Biol. Phys. *59*, 928–942.
7. Pasparakis, M., and Vandenabeele, P. (2015). Necroptosis and its role in inflammation. Nature *517*, 311–320.
8. Mills, K.V., Johnson, M.A., and Perler, F.B. (2014). Protein splicing: how inteins escape from precursor proteins. J. Biol. Chem. *289*, 14498–14505.
9. Topilina, N.I., and Mills, K.V. (2014). Recent advances in *in vivo* applications of intein-mediated protein splicing. Mob. DNA *5*, 5.
10. Wu, H., Hu, Z., and Liu, X.Q. (1998). Protein trans-splicing by a split intein encoded in a split DnaE gene of Synechocystis sp. PCC6803. Proc. Natl. Acad. Sci. USA *95*, 9226–9231.

11. Evans, T.C., Jr., Martin, D., Kolly, R., Panne, D., Sun, L., Ghosh, I., Chen, L., Benner, J., Liu, X.Q., and Xu, M.Q. (2000). Protein trans-splicing and cyclization by a naturally split intein from the dnaE gene of Synechocystis species PCC6803. J. Biol. Chem. *275*, 9091–9094.

12. Scott, C.P., Abel-Santos, E., Wall, M., Wahnon, D.C., and Benkovic, S.J. (1999). Production of cyclic peptides and proteins *in vivo*. Proc. Natl. Acad. Sci. USA *96*, 13638–13643.

13. Vila-Perelló, M., and Muir, T.W. (2010). Biological applications of protein splicing. Cell *143*, 191–200.

14. Kanno, A., Yamanaka, Y., Hirano, H., Umezawa, Y., and Ozawa, T. (2007). Cyclic luciferase for real-time sensing of caspase-3 activities in living mammals. Angew. Chem. Int. Ed. Engl. *46*, 7595–7599.

15. Kagiya, G., Ogawa, R., Hyodo, F., Yamashita, K., Nakamura, M., Ishii, A., Sejimo, Y., Tominaga, S., Murata, M., Tanaka, Y., and Hatashita, M. (2016). Development of a real-time imaging system for hypoxic cell apoptosis. Mol. Ther. Methods Clin. Dev. *5*, 16009.

16. Steel, G.G. (2001). The case against apoptosis. Acta Oncol. *40*, 968–975.

17. Zhuang, H., Shi, S., Yuan, Z., and Chang, J.Y. (2019). Bevacizumab treatment for radiation brain necrosis: mechanism, efficacy and issues. Mol. Cancer *18*, 21.

18. Mailloux, A., Grenet, K., Bruneel, A., Bénéteau-Burnat, B., Vaubourdolle, M., and Baudin, B. (2001). Anticancer drugs induce necrosis of human endothelial cells involving both oncosis and apoptosis. Eur. J. Cell Biol. *80*, 442–449.

19. Sancho-Martínez, S.M., Piedrafita, F.J., Cannata-Andía, J.B., López-Novoa, J.M., and López-Hernández, F.J. (2011). Necrotic concentrations of cisplatin activate the apoptotic machinery but inhibit effector caspases and interfere with the execution of apoptosis. Toxicol. Sci. *122*, 73–85.

20. Liu, H., and Baliga, R. (2003). Cytochrome P450 2E1 null mice provide novel protection against cisplatin-induced nephrotoxicity and apoptosis. Kidney Int. *63*, 1687–1696.

21. Megyesi, J., Safirstein, R.L., and Price, P.M. (1998). Induction of p21WAF1/CIP1/SDI1 in kidney tubule cells affects the course of cisplatin-induced acute renal failure. J. Clin. Invest. *101*, 777–782.

22. Ramesh, G., and Reeves, W.B. (2003). TNFR2-mediated apoptosis and necrosis in cisplatin-induced acute renal failure. Am. J. Physiol. Renal Physiol. *285*, F610–F618.

23. Ramesh, G., and Reeves, W.B. (2005). p38 MAP kinase inhibition ameliorates cisplatin nephrotoxicity in mice. Am. J. Physiol. Renal Physiol. *289*, F166–F174.

24. Fang, C., Wang, K., Zeng, C., Chi, C., Shang, W., Ye, J., Mao, Y., Fan, Y., Yang, J., Xiang, N., et al. (2016). Illuminating necrosis: From mechanistic exploration to preclinical application using fluorescence molecular imaging with indocyanine green. Sci. Rep. *6*, 21013.

25. Hall, M.P., Unch, J., Binkowski, B.F., Valley, M.P., Butler, B.L., Wood, M.G., Otto, P., Zimmerman, K., Vidugiris, G., Machleidt, T., et al. (2012). Engineered luciferase reporter from a deep sea shrimp utilizing a novel imidazopyrazinone substrate. ACS Chem. Biol. *7*, 1848–1857.

26. Shipunova, V.O., Shilova, O.N., Shramova, E.I., Deyev, S.M., and Proshkina, G.M. (2018). A Highly Specific Substrate for NanoLUC Luciferase Furimazine Is Toxic *in vitro* and *in vivo*. Russ. J. Bioorganic Chem. *44*, 225–228.

27. Kuchimaru, T., Iwano, S., Kiyama, M., Mitsumata, S., Kadonosono, T., Niwa, H., Maki, S., and Kizaka-Kondoh, S. (2016). A luciferin analogue generating near-infrared bioluminescence achieves highly sensitive deep-tissue imaging. Nat. Commun. *7*, 11856.

28. Iwano, S., Sugiyama, M., Hama, H., Watakabe, A., Hasegawa, N., Kuchimaru, T., Tanaka, K.Z., Takahashi, M., Ishida, Y., Hata, J., et al. (2018). Single-cell bioluminescence imaging of deep tissue in freely moving animals. Science *359*, 935–939.

OMTO, Volume *20*

Supplemental Information

Real-time visualization of intratumoral necrosis using split-luciferase reconstitution by protein trans-splicing

Go Kagiya, Ayaka Sato, Ryohei Ogawa, Masanori Hatashita, Mana Kato, Makoto Kubo, Fumiaki Kojima, Fumitaka Kawakami, Yukari Nishimura, Naoya Abe, and Fuminori Hyodo

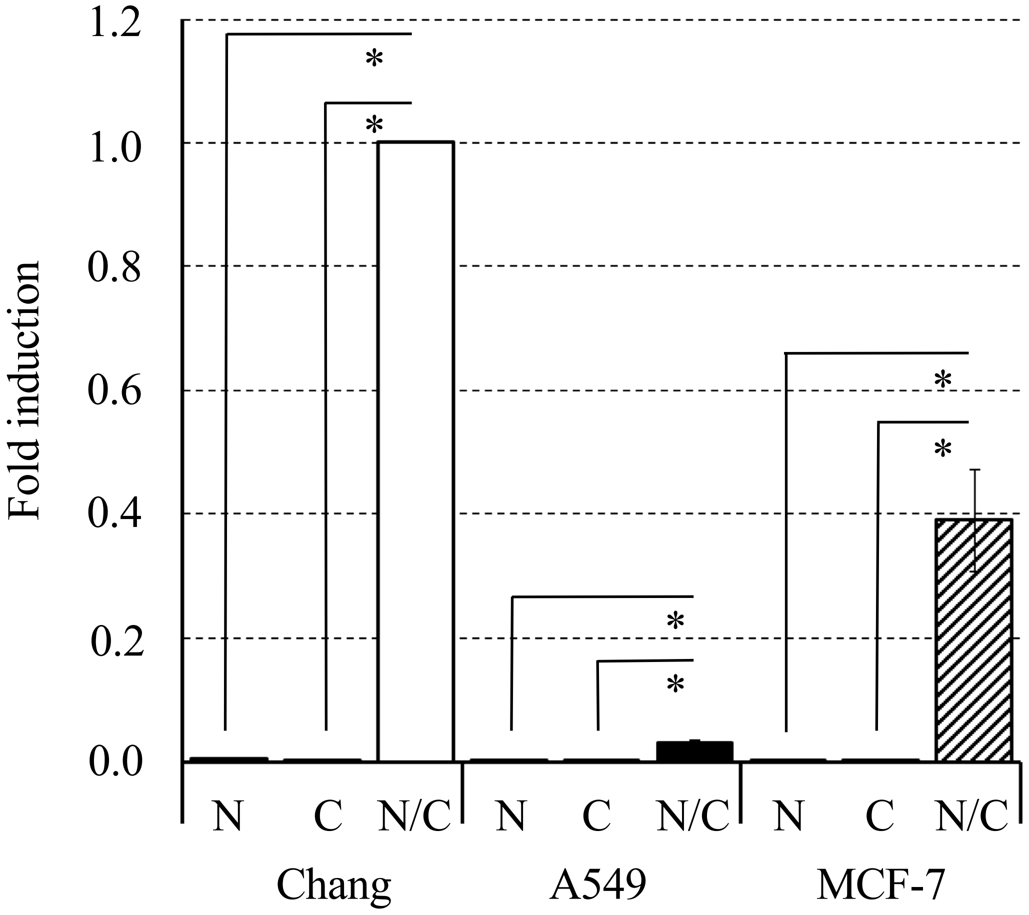

Figure S1. A comparison of the bioluminescence intensities obtained through the interaction of necrosis imaging reporters in three different cell lines. Chang liver cells, A549 cells and MCF-7 cells, transiently transfected with either pNLucN/v2 or pNLucC/v2-Fc were recovered at 12 h after transfection. The recovered cells were either lysed in a single transfectant or in a combination of the mutual transfectants of one cell line in PLB and then were incubated at an ambient temperature before undergoing the luc assay. Their bioluminescence values were normalized to those of the combined transfectant cell lysates with pNLucN/v2 and pNLucC/v2-Fc in Chang cells and then plotted as the mean fold induction. Error bars represent the standard deviation (n = 3). Asterisks indicate statistical significance ($p < 0.01$).

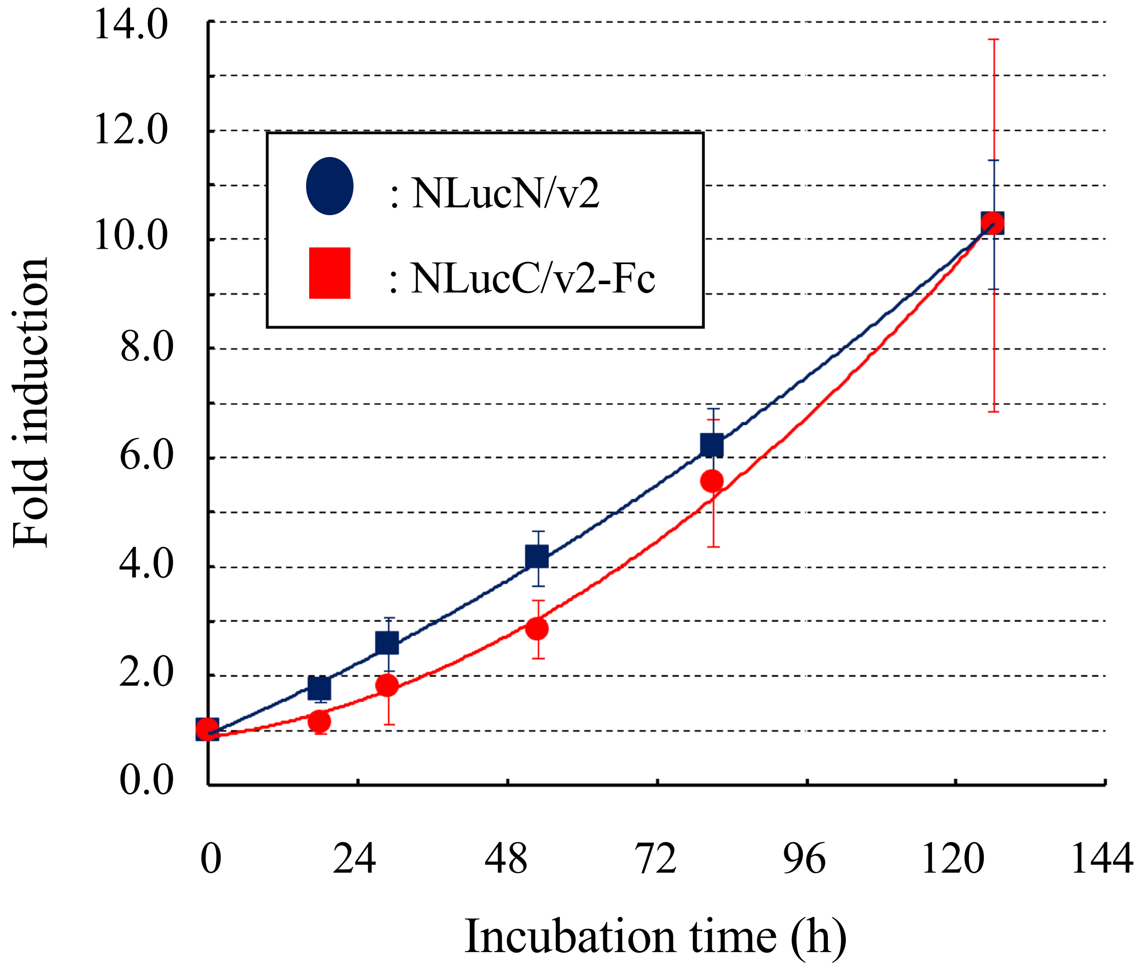

Figure S2. Cell proliferation curves of NLucN/v2 and NLucC/v2-Fc, which are stably transfected cell lines with pNLucN/v2 and pNLucC/v2-Fc, respectively. The cells inoculated in 96-well plate were pre-incubated at 37 ° C for 12 h, and then WST-8 was added to each well. The absorbance values obtained at 450 nm were normalized against those of the same cell line at the beginning point and then were plotted as the cell growth curve (n = 3).

Supplemental methods:

Cell proliferation measurement

The cell proliferation measurements were performed with the Cell Counting Kit-8 (Dojindo, Kumamoto, Japan) according to the manufacturer's instructions. Two necrosis imaging reporter cell lines, NLucN/v2 and NLucC/v2-Fc (2.0×10^3 cells/100 μl/well), were cultured in a 96-well plate for 12 h before 10 μl of WST-8, the colorimetric reagent of the kit, was added to each well and incubated at 37 ° C for 2 h. The absorbance value of each well was determined at 450 nm using a Multiskan FC micro-plate reader (Thermo Fisher Scientific, Waltham, MA, USA). Since the relationship between the cell numbers and absorbance at 450 nm after the addition of WST-8 was confirmed to be linearly correlated, the absorbance ratios normalized against those of the same cell line at the beginning point were plotted as the cell growth curve.
